# Dual branch fundus deep learning network as an enhanced multi classification system for ocular disease detection via hybrid feature fusion

**DOI:** 10.1038/s41598-026-58277-5

**Published:** 2026-07-02

**Authors:** Naglaa Samra, Hossam Moustafa, Mohamed Aouf, Waleed Abou Samra, Mohamed M. Abdelslam

**Affiliations:** 1 Department of Biomedical Engineering, Higher Technological Institute, 10th of Ramadan City, Egypt; 2 Department of Electronics and Communication Engineering, Faculty of Engineering, Mansoura University, Al Mansoura, Egypt; 3 Faculty of Artificial Intelligence and Information, Horus University, New Damietta City, Egypt; 4 Department of Ophthalmology, Faculty of Medicine, Mansoura University, Al Mansoura, Egypt; 5 Department of Computers and Control Systems Engineering, Faculty of Engineering, Mansoura University, Al Mansoura, Egypt; 6 Faculty of Engineering, Mansoura National University, Gamasa, Al Mansoura, Egypt; 7 Department of Biomedical Engineering, Faculty of Engineering, Mansoura University, Al Mansoura, Egypt

**Keywords:** Deep learning, OIA-ODIR dataset, Convolutional neural network, Feature fusion, Multi-classification, Computational biology and bioinformatics, Diseases, Mathematics and computing, Medical research

## Abstract

Ophthalmic diagnosis relies heavily on the interpretation of fundus images to identify a range of debilitating diseases. However, the presence of multiple, co-existing pathologies and the subtle visual cues associated with early-stage disease pose a significant challenge, necessitating the development of advanced diagnostic tools. We present DFD-Net (Dual-Branch Fundus Deep Learning Network), a deep learning solution that performs multi-label classification of various ocular diseases. The system achieves this by integrating the attribute maps from bilateral retinal photographs, like those from the right and left eyes. This study starts with a thorough pre-processing operation on the fundus images, represented in image rescaling, black border image cropping, contrast enhancement, and data augmentation of retinal photographs. Following this, the processing pipeline begins with a dual-branch feature extraction framework. A part of the system employs a ConvNeXt architecture to obtain complex semantic characteristics, while the parallel branch employs a U-Net encoder to capture fine-grained, structural characteristics across several scales. These complementary feature representations are then combined via a fusion mechanism. The fused feature representations are subsequently enhanced by a SENet Block for greater robustness through channel-wise recalibration. The resulting feature maps are reduced via Global Average Pooling, and then passed through a Dense layer for feature refinement. Finally, the features are classified by a Softmax output layer, consistent with the single-label multi-class formulation adopted in this work, to identify one of eight possible categories. The testing was implemented on the Ocular Disease Intelligent Recognition-Ophthalmic Image analysis (OIA-ODIR) dataset, which has retinal photographs that represent six distinct ophthalmic categories, macular degeneration, cataracts, glaucoma, hypertension, diabetic retinopathy, and myopia, images that present normal cases, and images that represent multiple other diseases, depicting eight different categories. The proposed DFD-Net system established precision, recall, F1- scores, and overall accuracy of 92.75%, 93.17%, 94.45%, and 93.77% on the On-Site testing collection and 91.50%, 93.19%, 92.11%, and 92.97% on the Off-Site testing collection, respectively. Ultimately, the suggested DFD-Net system proved high efficiency in precisely classifying multiple ocular diseases, offering a novel and outstanding approach for early detection of fundus diseases.

## Introduction

**Fig. 1:**
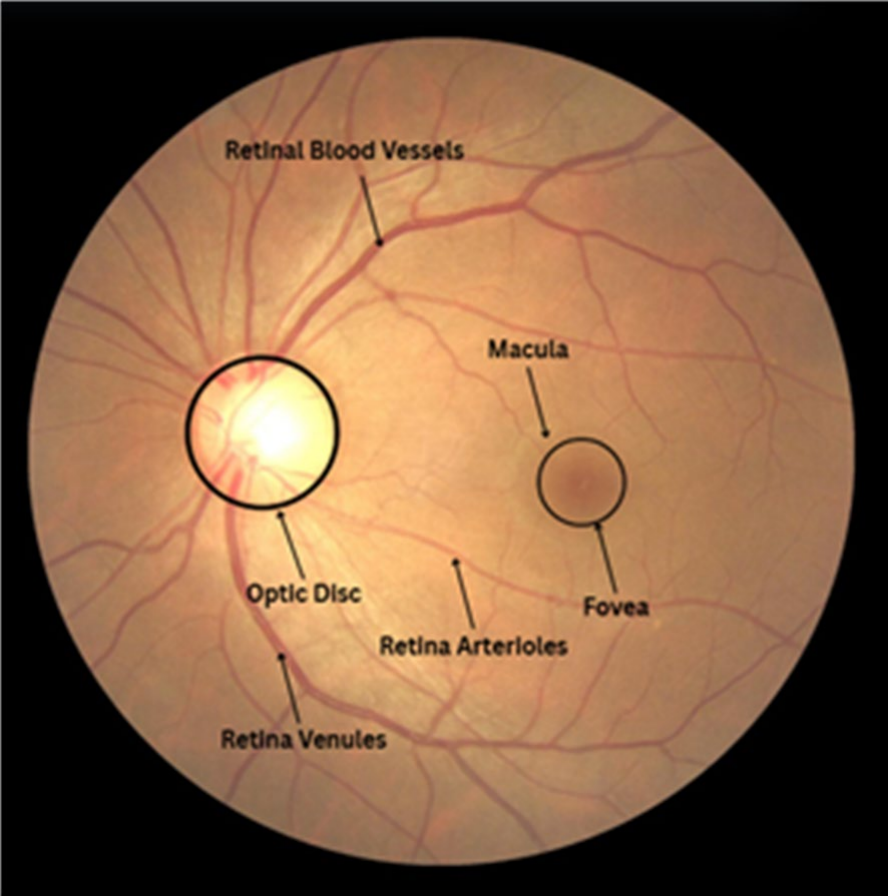
A normal fundus image from our dataset showing various regions of the retina.

According to the fact sheet on blindness and vision impairment updated by the World Health Organization (WHO) in 2023, a minimum of 2.2 billion individuals across the globe are living with a near or distance sight problem. Considering this population, estimates indicate that at least one billion cases, half of the total burden, could have been avoided or remain without necessary care. What chiefly causes this loss of distant sight and vision loss are age-related macular degeneration (8 million), cataract (94 million cases), glaucoma (7.7 million), diabetic retinopathy (3.9 million), and uncorrected sight impediment (88.4 million). Additionally, presbyopia is identified as the leading cause of loss of close sight, impacting 826 million people worldwide. Problems with vision or other health issues often become visible in the retina, which is a subtle tissue lining found at the rear of the eye’s inner chamber. It transforms incident light into electrical impulses that the occipital lobe processes to allow sight and perception of a scene or object^1^. It’s essential to spot eye diseases and treat them in their early stage to prevent lasting vision damage. Many of these eye diseases are difficult to detect early because they don’t have obvious symptoms. It’s also challenging for doctors to manually screen fundus images since these images contain complex anatomical details like retinal venules and arterioles, blood vessels, optic disc, and macula. This labor-intensive method is slow and often produces inconsistent results. That’s why Computer-Aided Diagnosis (CAD) is so important. CAD systems help doctors by rapidly and accurately analyzing these images, which saves time, reduces workload, and lowers costs. While traditional image analysis methods have their limits, the field has been fundamentally transformed by the recent emergence of deep learning. By design, deep learning systems independently learn and capture the most important features, including defects affecting the optic papilla, major blood vessels, macula, optic nerve head, and other features of the retina from the images, leading to more reliable diagnoses^2^. Figure 1 shows a labeled image of the normal human retina’s different parts.

Both fundus photography and optical coherence tomography (OCT) are established, effective methods for capturing ocular impairments in their stages. These imaging methods give ophthalmological experts a detailed look at the eye’s internal structures 3. OCT imaging provides a cross-sectional view of the eye’s layers, while fundus photography captures a wide, two-dimensional image of the retina. Fundus photography is a better option for general screening because it’s non-invasive and more affordable than OCT. The rise of digital retinal imaging has made it possible for doctors to provide remote consultations, making timely and accurate eye care more accessible, especially for people in remote or underserved areas^4^. In response to this, ocular photographs are the main route used by ophthalmological specialists to detect several ocular diseases. However, there are an assortment of reasons why using fundus images to diagnose ocular illnesses can be difficult. First of all, manually examining fundus photos demands significant time and energy, which makes it more difficult to arrive at a conclusive and definitive diagnosis^5^. There aren’t enough ophthalmologists in many underdeveloped nations to perform manual eye disease screenings. Furthermore, because the signs of common disorders including AMD, glaucoma, and diabetic retinopathy aren’t usually evident, it can be challenging to detect them early. Lastly, it can be difficult to obtain enough high-quality fundus photographs, especially for rare disorders. This is due to the fact that the images frequently lack contrast and include elements that could be interpreted as anatomical features. These problems make an automated Computer-Assisted Detection (CAD) system imperative. Derived from fundus photographs, such a technique would assist ophthalmologists diagnose patients quickly and precisely while also lessening their workload^2^.

Based on conventional, handcrafted feature extraction, numerous CAD systems have been introduced specifically to assist in diagnosing different ocular diseases. These approaches need a great deal of prior knowledge and have significant limitations. By way of example, Tamim et al.^6^ suggested a procedure for segmenting eye vessels utilizing a specific set of 24 hand-picked characteristics and a basic Multi-Layer Perceptron (MLP) neural architecture. Despite being tested on three public datasets (STARE, CHASE_DB1, and DRIVE), the system’s performance is only “comparable” to current techniques. The paper claims that this approach is valuable, but its complexity and the need for manual feature engineering make it a cumbersome and time-consuming option compared to more modern, automated deep learning methods. Deep neural networks (DNNs) have advanced significantly in computer vision over the last few years, outperforming more conventional handcrafted methods in this area^7,8^. Within the scope of medical imaging, Convolutional neural networks, a prominent inheriting class of DNNs, have shown noteworthy progress and efficiency. When performing procedures such as identifying objects or classifying diseases, these networks have shown exceptional efficacy. They are widely used in the diagnosis of ocular diseases, particularly in the detection of diabetic retinopathy stage classification^9,10^, glaucoma identification^11^, optic nerve segmentation^12^, optic disc segmentation^13^, and more, due to their capability to automatically pull out key distinguishing features from images. Given that CNN models have proven to be reliable for detecting fundus abnormalities, there are still certain restrictions and issues to be resolved. First off, the majority of published research has only examined a particular sort of eye diseases, as proposed in^9–11,14,15^. As a result, a lot of the models in use today perform well on particular tasks but could struggle to handle challenging real-world situations (such as classifying several fundus images). We assert the necessity of establishing a superior, more exhaustive fundus abnormality detection system designed to identify various ocular diseases concurrently. Likewise, there aren’t many datasets with real fundus photos labelled for various eye conditions. Subsequently, most established CNN algorithms classify fundus images using data from one eye, despite the fact that real-world clinical scenarios, ophthalmology specialists frequently use data from both eyes to make a diagnosis. Research has shown that the development of ocular disorders in both eyes is closely correlated^16^. This result suggests that a more efficient method of diagnosing ophthalmic patients would be to use data from bilateral fundus imaging. The diagnostic system’s overall performance can be improved in this situation by combining data from two fundus pictures using a variety of data fusion techniques^2^.

A swift, automated system using deep learning for classifying numerous categories simultaneously entitled DFD-Net (Dual-Branch Fundus Deep-learning Network), was created to tackle the problem of identifying several eye conditions in fundus images. The system receives paired fundus images, one from the right eye and one from the left eye, as input. It comprises three primary components. The first step is the implementation of an image pre-processing approach that comprises image rescaling, minimizing noise, removing the black border, enhancing contrast, and implementing data augmentation. Thereafter, a dual-branch framework used for attribute extraction receives a preprocessed pair of fundus images. This framework consists of a ConvNeXt backbone to extract high-level semantic features and a parallel U-Net encoder to extract fine-grained structural features. These individual extracted attributes are then merged to create a singular, consolidated data transformation. This unified feature map is then refined by a SENet Block, which improves the discriminative power of feature channels by recalibration. For the purpose of creating a probabilistic outcome profile over the eight ocular categories, the consolidated attribute is fed into a Global Average Pooling (GAP) layer and subsequently categorized by a dense layer with SoftMax activation. The principal contribution of this research is summarized below:

1. We present an efficient image enhancement pipeline to enhance contrast and reduce noise in ocular photographs. This method uses blue channel extraction to isolate vascular detail, contrast-limited adaptive histogram equalization (CLAHE) to increase regional contrast, and an unsharp mask to sharpen edges. We contend that deep learning models can significantly improve their ability to learn more valuable characteristic descriptions by being trained on this pre-processed data instead of raw images.
2. The study introduces an essential dual-branch attribute transformation framework to analyze paired left and right fundus images. It uses a ConvNext backbone for high-level semantics and a U-Net encoder for structural details. These are fused, refined by a SENet block for discriminative power, and then classified via GAP and SoftMax to yield a clear multi-label probabilistic outcome profile of eight eye conditions.
3. We show that the suggested DFD-Net system performs better than the current cutting-edge techniques. This assessment is carried out utilizing the OIA-ODIR dataset, a dataset that is publicly accessible that includes a wide range of different ocular photos covering eight different eye categories.

## Related works

This part shows a thorough analysis of the most innovative techniques for categorizing various ocular conditions, looking closely at their drawbacks, highlighting their key directions, and proposing potential solutions using our system’s architecture. Koh et al.^17^ presented an automated classification system for retinal diseases, including glaucoma, AMD, and DR, by using SURF and PHOG descriptors to extract discriminative feature representations. These features were then aggregated via the Canonical Correlation Analysis (CCA) technique, and a K-NN classifier was used for classification. Their dataset, which included1.804 ocular photographs, demonstrated a remarkable specificity of 97.42%, sensitivity of 95%, and accuracy of 96.21%. Islam et al.^18^ by employing pre-trained retinal images from the ODIR collection, the researchers developed a CNN model that underwent novel training; nevertheless, the model lacked the ability to concurrently classify multiple ocular pathologies using fundus image pairs. A Kappa score of 31%, an F1-score of 85%, and an AUC of 80.50% were among their finest results. Gour and Khanna^7^ utilized well-known pre-trained architectures, such VGG16, MobileNet, ResNet, and InceptionV3 for diverse ocular disease classification. The VGG16 model and SGD optimizer produced the highest results. On the OAI-ODIR dataset, they achieved an AUC of 85.57% and an F1-score of 84.93%. Yang and Yi^19^ created a model using deep learning with three components for the automated classification of several eye conditions. In the first step, the fundus image collection was artificially enlarged by implementing a basic image pre-processing technique to remove unnecessary information. They used the Xception architecture in the second section to implement an attribute extraction network, DSRA-CNN. The DS, DSR, and SE function blocks were all integrated into this network. Ultimately, eight different fundus categories were classified using a SoftMax classifier that was developed using the attributes that were retrieved. The created model obtained a Kappa score of 86.17%, F1-score of 88.16%, a precision of 88.50%, and an accuracy rate of 87.90% when tested on the OIA-ODIR collection. Al-Fahdawi et al.^2^ proposed the Fundus Deep-Net system, an automated technique using deep learning for detecting distinctive eye conditions utilizing pairs of ocular photographs. The system extracts rich feature representations using a High-Resolution (HR-Net) and an attention block, which are then refined and consolidated by an SENet block. A Discriminative Restricted Boltzmann Machine (DRBM) with a SoftMax activation unit is later utilized to categorize eight distinct eye conditions. The system’s effectiveness was demonstrated on the OIA-ODIR dataset, where it achieved an off-site test set F1-score of 88.56%, Kappa score of 88.92%, AUC of 99.76%, and final score of 92.41%, and on-site test set F1-score of 89.13%, Kappa score of 88.98%, AUC of 99.86%, and final score of 92.66%.

Through the examination of fundus photographs, the aforementioned research has shown the possibility and effectiveness of using deep learning-based techniques for the classification of certain eye conditions. Nonetheless, a few restrictions must be handled, such as: (i) Increased class cardinality results in degraded model performance, especially in scenarios characterized by scarce training data and unavoidable image corruption. (ii) Due to imbalanced or inadequate datasets, certain systems exhibit a bias toward caution, restricting their applicability in practical deployments. (iii) In the field of ocular disease classification, studies based on CNNs frequently employ unprocessed fundus photographs as the direct training data for their models, which can hinder the capability of the selected CNN model to efficiently manipulate or draw conclusions from the data. Our findings demonstrate that when the deep learning model is trained on processed ocular photographs, its generalization ability is shown to be greatly boosted, and its computational requirements are reduced compared to training directly with unprocessed image data. The DFD-Net system is an innovative multiple fundus diagnostic approach that overcomes the previous limitations and can accurately identify a variety of ocular disorders using colored retinal photographs. Our strategy involves deploying several augmentation techniques to artificially enlarge the training set, thereby addressing overfitting and data scarcity. Additionally, for the purpose of improving generalization and preventing model over-optimization, the proposed DFD-Net architecture was trained exclusively on the pre-processed fundus images, bypassing the direct use of raw image data.

## Methodology

**Fig. 2:**
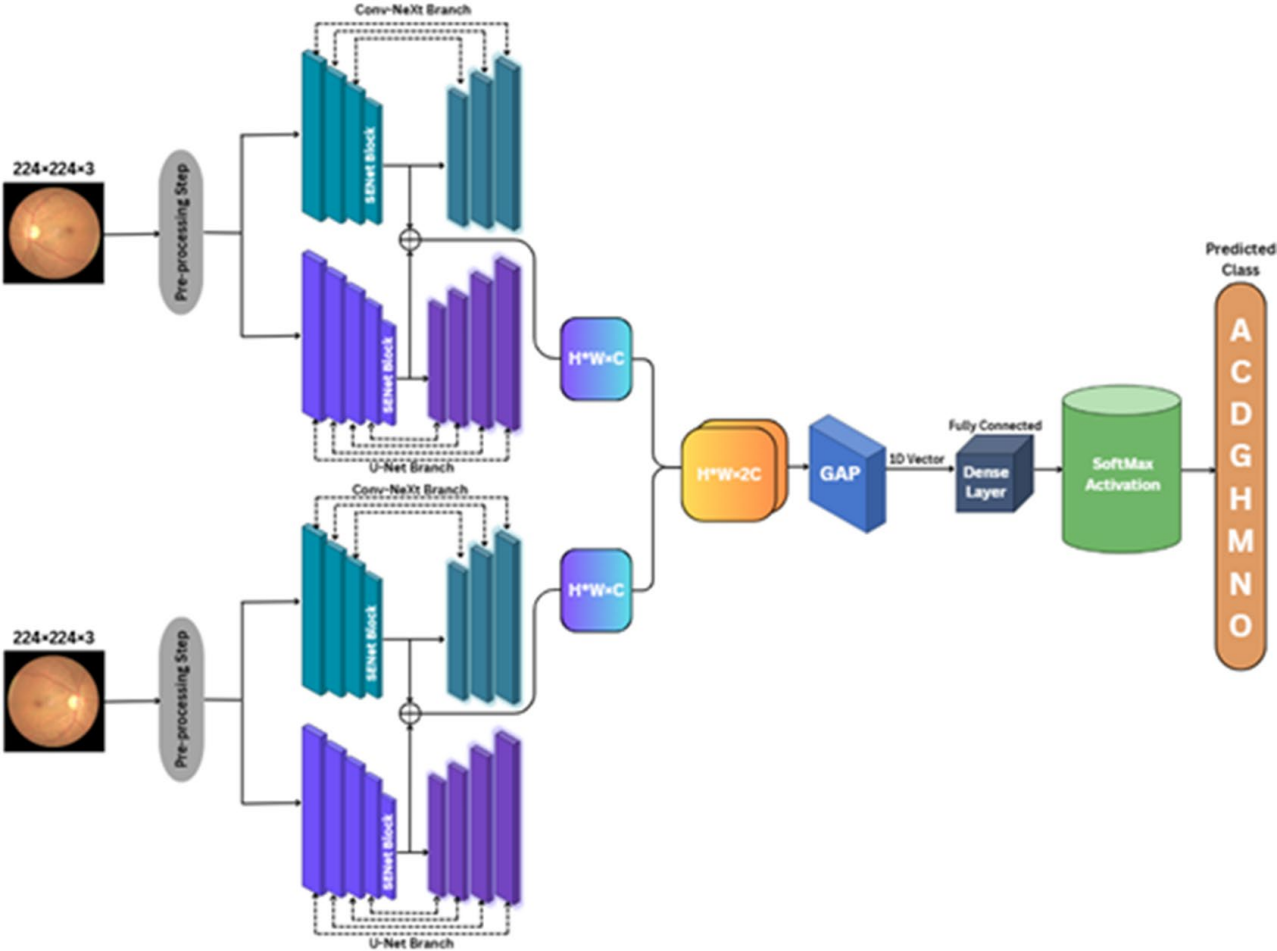
The block diagram representing the structure of the proposed DFD-Net system.

The architecture of the DFD-Net system, designed to sort fundus images into eight distinct categories of eye conditions, is presented in Fig. 2. The system is introduced to tackle the challenge of identifying multiple eye conditions within fundus photographs. Three main methodological components make up the proposed DFD-Net System. First, a thorough custom preprocessing pipeline including Blue Channel Extraction, Contrast Limited Adaptive Histogram Equalization (CALHE), then the input fundus photographs undergo Unsharp Masking. This sequence is utilized to enhance image contrast, mitigate noise, and accentuate structural features crucial for pathology discrimination. Second, a robust dual-branch deep feature extraction and fusion architecture receives the preprocessed image. Each eye image is processed independently through the same dual-branch network, producing two separate fused feature maps: IR for the right eye and IL for the left eye. These two feature maps are subsequently fed into the SENet block, where they are combined via channel-wise concatenation to produce the final unified feature representation Fc. This approach uses a ConvNeXt Backbone (pre-trained and frozen) to extract robust, high-level semantic features through transfer learning, while a U-Net Encoder route captures multi-scale, fine-grained spatial information. The backbone architecture is used just as a multi-scale feature extractor, even though it has an encoder-decoder structure that was modified from BCU-Net^20^. The decoder allows the network to record fine-grained spatial characteristics at several resolutions, which are then sent to the classification head without producing pixel-wise segmentation output. A concatenation technique is utilized at the bottleneck layer to combine these two different feature representations, creating a highly detailed fused feature map. Finally, Global Average Pooling (GAP) is applied to this fused representation, which then goes through a dense classification head that results in a SoftMax activation function. The final diagnostic categorization is produced by this technique, which creates the probability distribution across eight target ocular illness categories. This architecture ensures excellent feature fusion for high-fidelity illness prediction by leveraging the proven feature hierarchy of the ConvNeXt backbone and the structural localization strengths of the U-Net encoder.

## Dataset

**Fig. 3:**
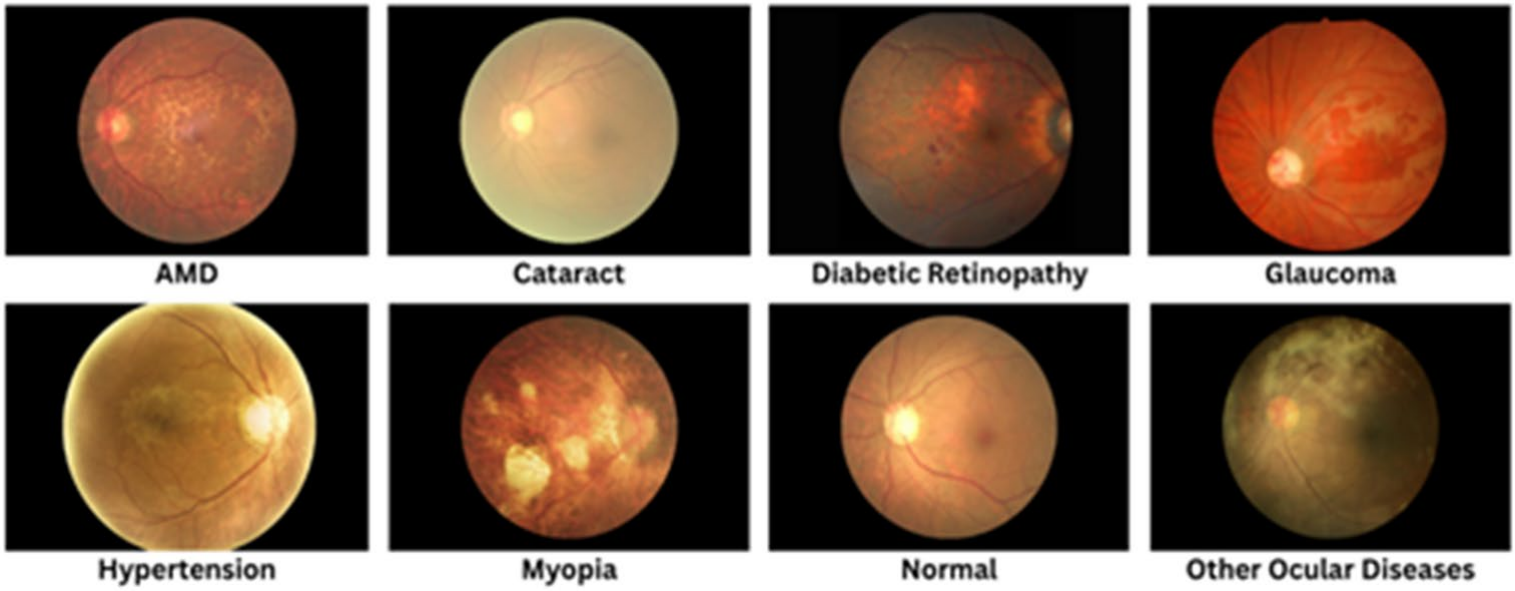
Some samples of fundus photographs from the OIA-ODIR.

This study evaluated the DFD-Net system’s accuracy using a number of comprehensive tests conducted on the OIA-ODIR dataset^5^. The first global dataset for the detection of multiple eye conditions using fundus images was produced by Shanggong Medical Technology Company and is made publicly available. It includes 10,000 retinal photographs from 5000 medical cases, each of whom represents a class of eight different ocular conditions. A training collection comprising 3500 medical cases, an off-site test set comprising 500 patients, and an on-site test set comprising 1000 medical cases comprised the three subsets of the dataset. The training set is used to train deep networks, while the testing is done on both the off-site collection and the on-site collection. Eight categories are included in the multi-class OIA-ODIR dataset for the detection of retinal diseases: Age-Related Macular Degeneration (A), cataracts (C), diabetic retinopathy (D), glaucoma (G), hypertension (H), myopia (M), normal (N), and other ocular conditions (O). It is worth noting that while the dataset accommodates multi-label cases, each patient record is associated with one predominant diagnostic label, which supports the single-label multi-class treatment adopted in this study. Table 1 represents the division of the dataset between the training and testing collections. It took more than ten months for skilled ophthalmologists to annotate the dataset with ground truth. Discrepancies were settled via discussion until a unanimous agreement was reached among all annotators^5^. Figure 3 illustrates select samples drawn from the OIA-ODIR collection.

It is important to note that the standard benchmark release assigns a single predominant diagnostic label per patient record, despite the OIA-ODIR annotation schema recognizing the potential for co-existing illnesses. The multi-class classification formulation used in this study, for which Softmax activation is the proper and conventional option, is supported by this single-label structure, which is consistent with other work on this dataset.

**Table 1:** Case counts stratified by class within the training and testing data collections.

Classes	*N*	D	G	C	A	H	M	O
Training group	1138	1130	215	212	164	103	174	982
Off-site testing group	162	163	32	31	25	16	23	136
On-site testing group	324	327	58	65	49	30	46	275
Total	1624	1620	305	308	238	149	243	1393

### Data preparation phase

The retinal photographs within the OIA-ODIR collection exhibit substantial variability across key characteristics, including lighting, resolution, color saturation, and image contrast. Pre-processing techniques may be required to tackle these issues and pose a challenge to image analysis algorithms^5^. The suggested pre-processing phase is implemented in five major stages: image rescaling, black border cropping, feature isolation, feature enhancement, and finally, data augmentation. In the OIA-ODIR dataset, the preponderance of the fundus photographs contain black borders that fail to include the essential data required to identify eye conditions (See Fig. 3). Removing the dark, unnecessary circular borders lessens the black borders’ detrimental effects. The automatic smart cropping process or Region of Interest (ROI) Extraction in our experimentation is carried out as follows:

1. Representation of ocular photographs in the utilized dataset in RGB format into greyscale using the OpenCV library. The white color is set to a value of 225 and 0 for pixels that represent the color black.
2. A binary mask is created by thresholding the grayscale image. A value of 1 is assigned to every pixel value that exceeds a tolerance value that is set by default to 6, while all other pixels are clamped to 0. The brilliant retinal disc and the dark backdrop are successfully separated by this technique.
3. Implement a rectangle that has four corners representing the highest, lowest, leftmost, and rightmost points of pixels’ value set to 1, which in our project is the retina. This rectangle defines the precise coordinates needed for the final crop.
4. The coordinates of the rectangular region are applied to the original, three-channel image data to localize the area of interest, effectively producing a precisely cropped and centered RGB photograph with the minimal surrounding black background.

**Fig. 4:**
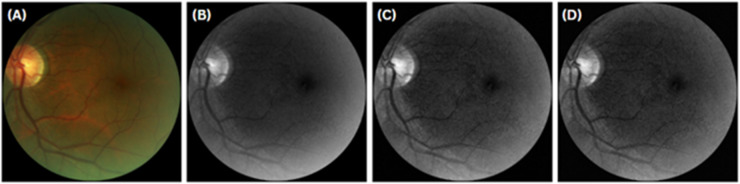
The results of the proposed pre-processing technique: (**A**) Original fundus image after cropping, (**B**) Applying blue channel extraction, (**C**) Applying CLAHE, (**D**) Applying unsharp masking.

This first crop is essential because it reduces computing time by eliminating superfluous black padding and increases accuracy by guaranteeing that only the pertinent retinal data is used in all subsequent analyses. Additionally, because they were taken using different cameras, the foundational images in this dataset vary in size. Expectedly, the following step is to standardize the dimensions of the clipped image to a size supported by most of the DNN models (224 × 224). Next, the contrast of vessels and lesions is maximized using the Blue Channel Extraction technique^21^. Only the blue channel’s pixel values are extracted from the RGB input image to create a single-channel grayscale image. Blood vessels, hemorrhages, and microaneurysms exhibit the greatest light absorption in the blue spectrum. By isolating these darker features, this process suppresses the background’s reddish hue while generating the best signal-to-noise ratio for diagnostic structures. As presented in Fig. 4, blue channel extraction is followed by applying Contrast Limited Adaptive Histogram Equalization (CLAHE)^22^. Instead of global processing, CLAHE adaptively enhances contrast within localized tiles. Bilinear interpolation is then applied to the tile boundaries to seamlessly blend them and suppress spurious artifacts. The contrast of the image improved by CLAHE is managed by two crucial parameters. A greater value for the CL factor results in an improvement in the brightness of the input data because of its small intensity degree. On the other side, raising the BS parameter value broadens the input image’s range of intensity values and enhances the contrast level. After experimentation, the BS and CL values in this investigation were set to (8 × 8) and 2, respectively.

**Fig. 5:**
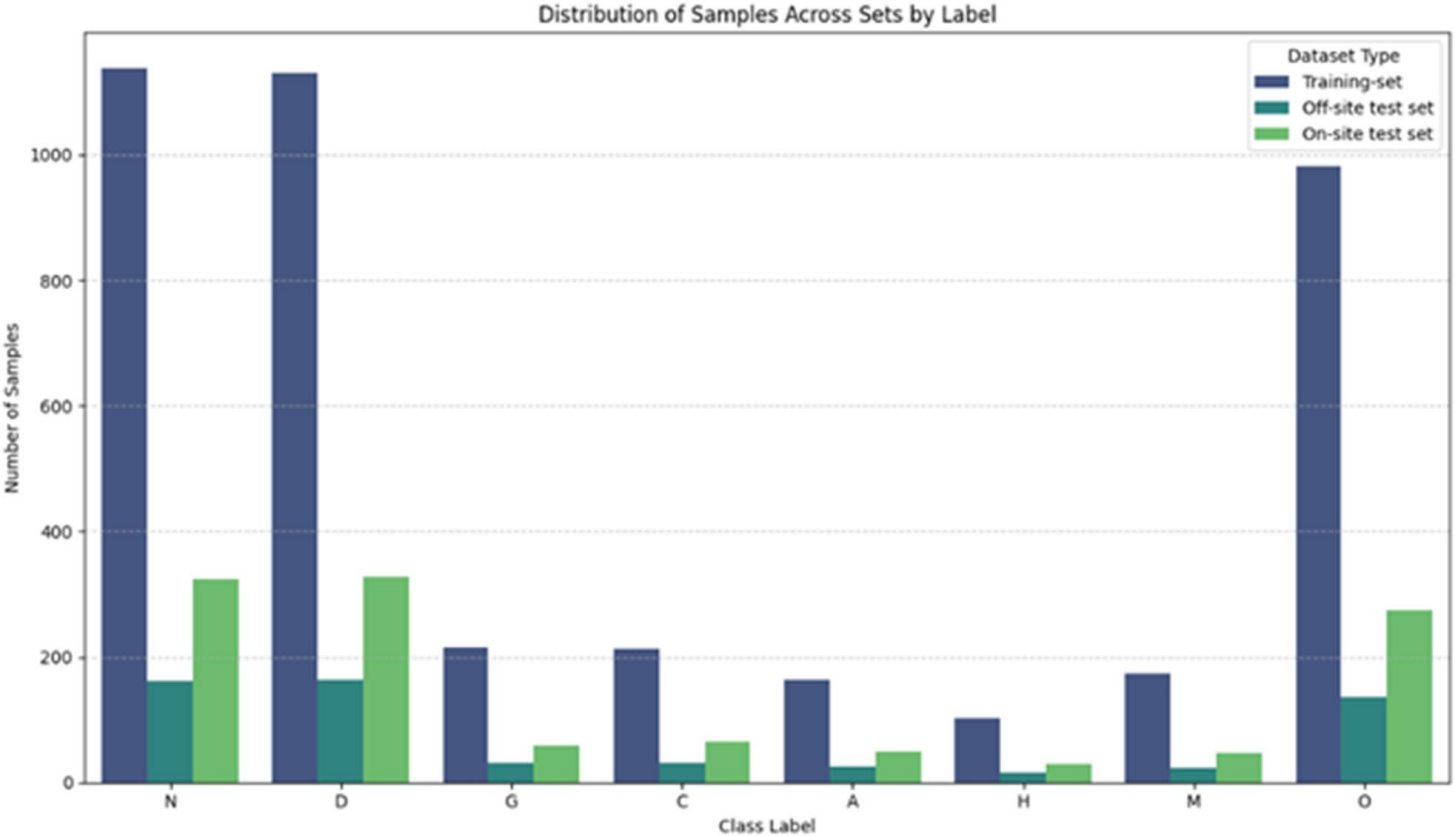
Sample distribution of the OIA-ODIR dataset.

Resulting in the improvement of the contrast of fine vascular borders and faint lesions in darker areas while avoiding over-amplification of noise in areas of low variance (homogeneous background). This guarantees that, despite local lighting fluctuations, features are consistently visible throughout the retinal image. And for further edge sharpening for the vascular and pathological structures and segmentation precision improvement, we use the Unsharp Masking (USM) method^23^. Kernel size (ksize), amount, and threshold are key parameters that control how aggressively the image is sharpened. Ksize defines the size of the blur used to create the unsharp version of the image (mask). A moderate size of Ksize is a good compromise, targeting mid-size features while avoiding excessive noise amplification. Amount or strength controls the intensity of the sharpening by determining how much of the detected sharpness is added back to the original image. Threshold is an optional but critical parameter. It sets a minimum contrast value that an edge must have before the sharpening is applied. A small, non-zero value is used to ensure that only true structural edges are sharpened, and background noise is ignored. The ksize, amount, and threshold in this investigation were set to (5 × 5), 1.2, and 4, respectively, (See Fig. 4). The classes of the OIA-ODIR dataset show great imbalance as shown in Fig. 5, due to the uneven distribution of photographs across categories, those classes with the greater number of samples will be assigned greater training weights than smaller classes. This approach risks biasing the classification results in favor of the more populous categories.

For example, some categories contain fewer than 200 fundus images, such as hypertensive retinopathy, myopia, and AMD, in the training set, whereas there are 1138 normal classes. Some data augmentation approaches, including rotation, horizontal and vertical flipping, adjutments in saturation, hue, and brightnes, were done to address class imbalances and avoid the overfitting issue. Specifically, the augmentation pipeline was configured with a rotation range of ± 30°, horizontal and vertical flipping, a shear range of 0.2, a zoom range of 0.3, and a brightness range of [0.5, 1.5]. All augmented images were padded with a constant fill value of 0 to preserve the retinal region of interest.

## Backbone networks

For our backbone model, we chose a high-performance dual-branch feature extraction model that bridges between U-Net and ConvNeXt for these four purposes:

1. The ConvNeXt branch focuses on the global pathological semantics using the model’s extensive interaction capabilities.
2. The U-Net branch handles detailed processing, so it is used to focus on local pathological semantics.
3. Merging the unique strength of these two heterogeneous networks smartly and conveniently derives a richer, more complete set of features than either network could provide alone, which extracts sufficient complementary pathological semantics.
4. The efficient integration of the two distinct branches is particularly effective at mitigating the inherent class imbalance issue within the multi-classification task.

**Fig. 6:**
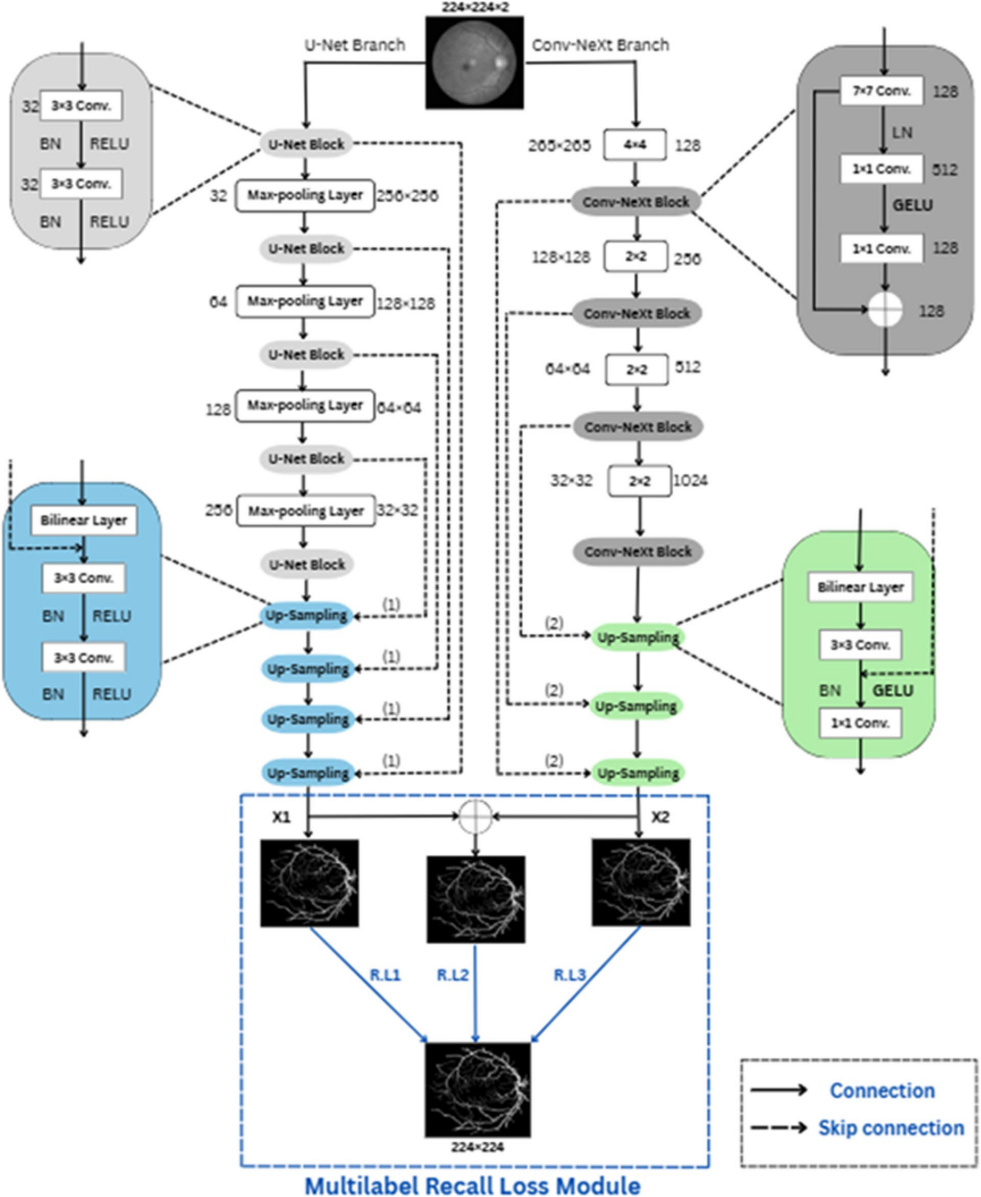
The parallel ConvNeXt Branch and U-Net Branch are displayed in the DFD-Net System’s Dual-Branch Backbone Structure.

As represented in Fig. 6, our backbone network consists of two branches. The standard ConvNeXt branch was not used, as it lacks the U-shaped convolutional structure needed for multi-scale spatial feature extraction; therefore, we adopted the procedure suggested by H. Zhang et al.^20^, extending the original encoder structure by adding a specific decoder component to capture fine-grained features at varying spatial resolution. Meanwhile, the U-Net branch streams a smaller contextual scope, focusing on local pathological semantic extraction. The synergy is completed when the ultimate output representation from each branch is fused via the Multiable Recall Loss block, allowing the heterogeneous networks to combine their strengths for robust multi-scale feature representation in support of disease classification. Prior to concatenation, the spatial resolution of the ConvNeXt feature map is aligned with that of the U-Net feature map via bilinear interpolation, while a 1 × 1 convolution is applied to match the channel dimensions of the two branches, ensuring a seamless and dimensionally consistent fusion at the bottleneck layer.

First, the auxiliary U-Net Branch’s architecture closely resembles the traditional U-Net design in order to concentrate on capturing local, fine-grained characteristics. The U-Net stream is structured with five downsampling layers, forming the encoding stage. Each of these blocks (in the U-Net branch) is composed of a pair of consecutive convolutional layers, each utilizing a 3 × 3 kernel, followed by a one Batch Normalization (BN) block^24^, and a Rectified Linear Unit (ReLU) block. The U-Net branch also contains four skip connections between the five downsampling blocks of the encoding phase and the four upsampling blocks of the decoding phase, each upsampling block (Up-Sampling [1]) contains a single bilinear layer, a pair of sequential convolutional layers. Each utilizing a 3 × 3 kernel, a single BN block, and a single ReLU block. The U-Net stream creates the ultimate output representation, X_**1**_, which is then supplied to the MRL module for integration of features. The convolutional layer utilizing the smaller kernel size in the suggested model is adequately able to capture local pathological semantics, thanks to the U-Net stream.

Second, the ConvNeXt stream is modified by adding a feature refinement stage to enable the extraction of spatially-aware, multi-scale representations from fundus photographs. Hence, the ConvNeXt stream comprised four sequential downsampling layers, each downsampling layer in the ConvNeXt Block is constructed from a (7 × 7) convolutional layer, a LayerNorm (LN), a single convolutional layer utilizing a 1 × 1 kernel, a Gaussian-based non-linear unit^25^, and a final 1 × 1 convolutional layer. The ConvNeXt branch has 3 skip connections between the encoding phase and the and the 3 upsampling blocks of the decoding phase, each upsampling block (Up-Sampling [2]) contains a convolutional layer utilizing a 3 × 3 kernel, a BN block, a GELU block, and finally a convolutional layer utilizing a 1 × 1 kernel. The ConvNeXt branch produces a feature map, X_**1**_, which is subsequently forwarded to the MRL module, where deep-level features are combined.

The process concludes with the MRL module, which enables a profound integration of both local and global semantics across the two streams. Furthermore, the Recall Loss function specifically assigns weights to a class’s samples based on their ability to instantly recall information. To account for the relative performance differences among classes, the associated weights were dynamically changed. While the gradients corresponding to the majority classes are slightly suppressed, the gradients for the minority classes are amplified. The utilized loss function combines Cross Entropy (CE) loss with a weighting factor according to the recall performance of each class^20^. The loss function is implemented as:

1 $$\:RecallCE=-\sum\:_{c=1}^{C}\left(1-\frac{{TP}_{c}}{{FN}_{c}+{TP}_{c}}\right){\times\:N}_{c}\times\:\mathrm{log\:}({P}^{c})=\:\sum\:_{c=1}^{C}\sum\:_{n:{y}_{i}=c}\:{log}\:({P}_{n})\times\:(1-{R}_{C})$$

where $$\:\{{x}_{n},\:{y}_{n}\}\forall\:n\:\in\:\:\{1,\:\dots\:,\:N\}$$ and $$\:{x}_{n}$$ represent the input training images, *and*$$\:\:{y}_{n}\:\in\:\:\{1,\:\dots\:,\:C\}$$ represent the ground truth labels. In this study, $$\:C\:=\:8$$. *N*_*C*_ represents the pixel count in category *C.* $$\:c\in\:\:\{1,\:\dots\:,\:C\}.\:{P}^{C}\:={\left(\prod\:_{n:{y}_{n}=c}{P}_{n}\right)}^{\raisebox{1ex}{$1$}\!\left/\:\!\raisebox{-1ex}{${N}_{c}$}\right.}$$ quantifies the average confidence using the geometric mean associated with category *C*. $$\:{P}_{n}\:$$ represents the model’s confidence scores, output via the SoftMax function, for classifying the input image $$\:{x}_{n}$$ into each available class. $$\:{TP}_{C}\:$$quantifies the count of true positives for category *C*. $$\:{FN}_{C}$$ quantifies the count of false negatives for category *C*. $$\:{R}_{C}$$ represents the class-specific recall score for category *C*.$$\:\:\{n\::\:{y}_{i}\:=\:c\}$$ represents the collection of images that are assigned to category *C*. Unlike standard Weighted Cross-Entropy, which assigns fixed, static weights to each class prior to training based solely on the class distribution, RecallCE dynamically adjusts the weight of each class during training based on the model’s actual recall performance. Classes exhibiting poor recall receive progressively amplified gradients, while majority classes receive suppressed gradients, ensuring continuous adaptation of the learning focus throughout training.

As illustrated in Fig. 6, the two branch design has three recall losses, $\:{R.L}_{1},\:{R.L}_{2},\:and\:{R.L}_{3}$, that are combined together by simple addition as shown in Eq. (2) during the training phase, whereas $\:{R.L}_{2}$ supervises the fused feature representation during the test phase.

2 $$\:Total\:Loss={R.L}_{1}+R.{L}_{2}+R.{L}_{3}$$

The $\:{R.L}_{1}$is responsible for the U-Net branch, while $\:{R.L}_{3}$ is responsible for the ConvNeXt branch. The total loss utilizies RecallCE as shown in Eq. (1). We can simply state that $\:{R.L}_{2}\:$is a bridging technique after the high-abstraction integration of $\:{X}_{1}$ and $\:{X}_{2}$. The $\:{R.L}_{2}\:$ recall loss is represented in the next formula:

3 $$\:{R.L}_{2}=a{R.L}_{1}+b{R.L}_{3}\:$$

The factors a and b serve as the numerical weights of $\:{R.L}_{1}$ and $\:{R.L}_{3}$ loss values, respectively.

### SENet block

**Fig. 7:**
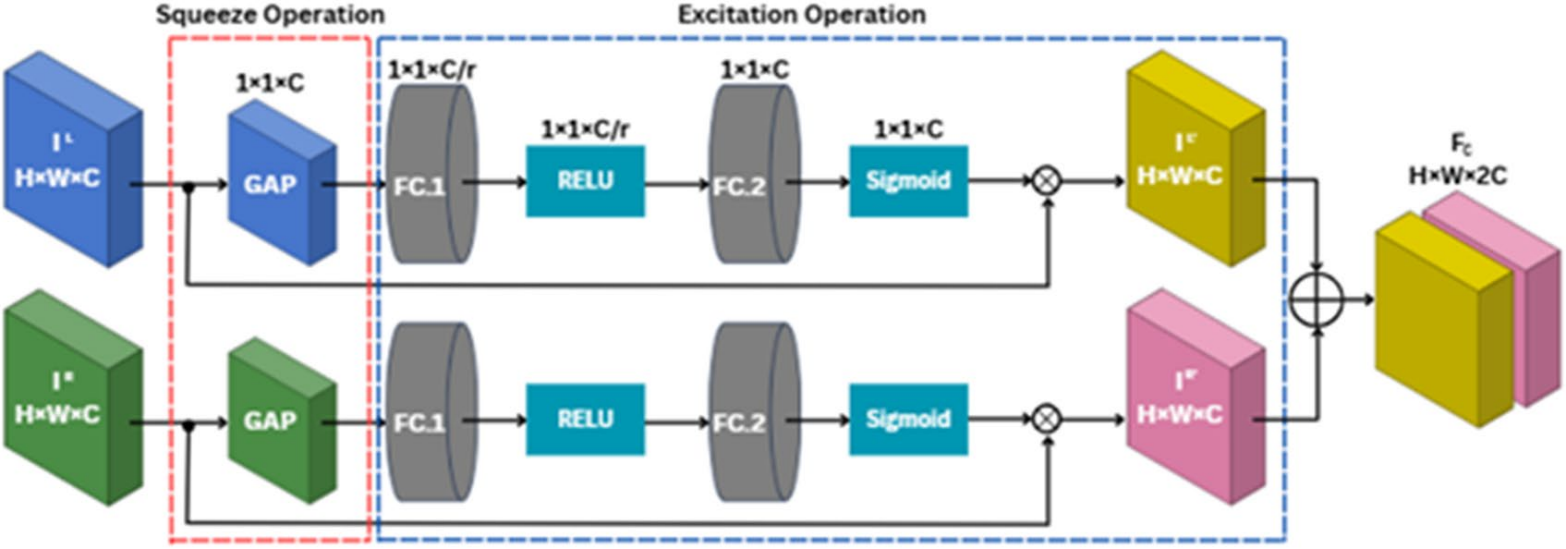
The main structure of the utilized SENet block.

To improve network performance, a channel attention method known as SENet^26^ can be taught to highlight important feature representations and suppress those that aren’t relevant. As a result, the suggested DFD-Net system now incorporates SENet. Figure 7 shows the main structure of the used SENet block. Before being fed into the subsequent transformation, a SE block receives holistic feature data in a couple of stages: a squeeze process and an excitation process. Squeeze operations are used to extract holistic positional information into a channel descriptor by producing channel-wise holistic features $\:1\:x\:1\:x\:C$ using two-dimensional Global Average Pooling (GAP). The purpose of the excitation operation is to employ a chain involving two fully connected layers, a non- linear ReLU, and a final sigmoid activation to aggregate the information from the squeeze operation. We start by reducing the dimension using an FC layer with a compression rate of *r* in order to restrict the complexity of the model. After experimenting with different values of *r*, we conducted that increasing *r* improves the recognition performance of fundus diseases, on the other side, aggressive increasing of the reduction ratio can lead to the generation of feature redundancy as well as boosting the model’s capacity. The best results were achieved when $\:r\:=\:14$. Subsequently, the architecture’s ability to model non-linear functions was enhanced by employing a ReLU block. Following this, the channel dimension is reinstated using a Fully Connected (FC) block, which functions as a layer for increasing dimensionality. The weight of each channel feature is then changed using a Sigmoid activation to improve the feature’s distinguishability. The excitation stage sequentially lowers and then increases the feature dimensionality as a technique to minimize network calculations while simultaneously enhancing the non-linear ability of the model to capture inter-channel dependencies. Multiplication of the incoming feature representation that resulted from the fusion of the ConvNeXt stream and the U-Net stream of the right eye $\:{I}^{R}:\:(H\times\:W\times\:C)$, and the feature map that resulted from the fusion of the ConvNeXt stream and the U-Net stream of the left eye $\:{I}^{L}:\:(H\times\:W\times\:C)$ ,with the sigmoid of each channel individually to yield the resulting, refined feature representation $\:{F}_{c}:(H\times\:W\times\:2C)$, as shown in Eq. 4, is the final operation in the SENet block, which focuses on representational ability improvement of the network.

4 $$\:F_{c} = I^{{R^{\prime } }} \oplus I^{{L^{\prime } }}$$

As shown in Fig. 2, the SENet block is implemented within the down-sampling sequence (encoders) of the U-Net stream and the ConvNeXt stream to ensure that the channels containing the highest diagnostic information are amplified and passed down to the deepest bottleneck fusion point, whereas channels containing less essential information are suppressed, leading to more robust classification outputs.

### Classification head

The Classification Head, the last part of the architecture, is in charge of converting the deep, fused feature map into a probabilistic prediction. Next, a Global Average Pooling (GAP) layer processes the fused map after the concatenation of features from the ConvNeXt Backbone and the U-Net Encoder^27^. By calculating the mean feature value over the whole spatial extent, this crucial step significantly reduces spatial dimensionality and produces a 1D discriminative feature vector. In order to improve feature separability in the final decision space, this feature vector is subsequently fed into a sequence of Fully Connected (Dense) layers, which act as non-linear mapping functions. The output layer is made up of a Dense layer with $\:K$ units, where $\:K$ is the number of classification categories. This layer yields the raw prediction scores, known as logits $\:({Z}_{1},\:{Z}_{2},\:\dots\:..,{Z}_{K})$. Since each patient in the OIA-ODIR benchmark is assigned a single predominant diagnostic label, the classification task is formally defined as a single-label multi-class problem. Accordingly, the Softmax activation function is applied to the output layer, producing a normalized probability distribution across the eight target classes. Finally, the Softmax activation function $\:\left(\sigma\:\right)$ is applied to these logits (see Eq. 5):

5 $$\:{\sigma\:\left(Z\right)}_{i}={P}_{i}=\frac{{e}^{{Z}_{i}}}{\sum\:_{j=1}^{K}{e}^{{Z}_{j}}}$$

6 $$\:\sum\:_{i=1}^{K}{P}_{i}=1$$

Although the OIA-ODIR dataset supports multi-label annotations, analysis of the label distribution reveals that the overwhelming majority of patients carry a single dominant diagnosis. Consistent with prior work on this dataset^2,19^, we therefore treat the classification task as a single-label multi-class problem, for which Softmax activation with categorical cross-entropy loss is the appropriate and standard choice. The Softmax activation function transforms the logit scores into a normalized probability distribution $\:P=\{{P}_{1},\:{P}_{2},...,\:{P}_{K}\}$, as illustrated in Eq. 6, the summation of all probablistics must equal 1, allowing the model to determine the probability of the incoming image corresponding to each of the 8 specified fundus disease classes. The loss function used for multi-classification, which measures the difference between the predicted distribution and the ground-truth distribution, is used to train this model^28^.

## Experimental results

Comprehensive information about the implementation of every experiment carried out is provided in this section. Additionally, it describes the evaluation metrics for the suggested DFD-Net system. Subsequently, the DFD-Net system’s dependability and effectiveness are assessed by contrasting it with other advanced, well-established systems.

### Implementation details

The DFD-Net architecture is written in Python using an A100 GPU and was developed using a Google Colab environment. The underlying hardware included a 69 K GPU graphics card, 16 GB of RAM memory,, and an Intel Core i7–4810MQ CPU, operating on a 64-bit Windows 10 OS. The deep learning architectures utilized in this study were implemented with TensorFlow. For consistency across the experiments, the original photographs were scaled down to a standard resolution of (224 × 224). Since many DNNs accept this standard size as the typical picture dimension, it was adopted. The OIA-ODIR dataset comes from several hospitals with various types of cameras, necessitating standardization. The dataset for this study was divided into training and testing groups at a 7:3 ratio (8000 photographs for training and the remaining 3000 photographs are assigned for testing). The training data was then subdivided into 80% of the original training group from the OIA-ODIR dataset (6400 samples), which was used for direct training, and the remaining 20% (1600 samples) served as a dedicated validation group. The DNNs used in this experiment were trained using the Adam optimization algorithm, with a batch size of 32 samples, an initial learning rate of 0.001, a weight decay of 0.0005, a dropout ratio of 0.5, and a momentum of 0.9. Additionally, the number of necessary training epochs for each DNN was determined using an early stopping strategy. This technique autonomously halts training when the system detects an upward trend in the validation set’s classification error rate, preventing overfitting. A constant of 100 epochs is used in all of the tests carried out in this study.

### Evaluation metrics

We calculated six quantitative performance measures to assess the efficacy of the suggested DFD-Net system on the OIA-ODIR collection: overall accuracy (OR), weighted precision (PR), weighted recall (RE), weighted F1-score (F1), Kappa score (KS), and Area Under Curve (AUC). The primary assessment statistic for the classification task is OR, which is the ratio of correctly classified examples (TPs and TNs) to the total number of instances experimented (See Eq. 7). Precision, also known as the Positive Predictive Value (PPV), measures how accurate or confident the model is in its positive claims. It is calculated as the ratio of TP predictions to the total number of cases classified as positive (TP + FP, FP) (See Eq. 8). Recall is also known as sensitivity or true positive rate (TPR). It is calculated by dividing the total number of real positive cases (TP + FN, FN) by the TP forecasts. It measures how comprehensive the model is or how well it can find all pertinent examples (See Eq. 9). The F1-score is a single metric calculated as the harmonic mean of both recall and precision. Its value effectively represents a balance between completeness (recall) and accuracy (precision) to provide a reliable assessment of a model’s performance. When looking for an even distribution of performance across both criteria, it is especially helpful (See Eq. 10). Cohen’s Kappa (k) Score is responsiable for the inter-rater reliability, or in machine learning, the degree of agreement between the model’s predictions and the ground truth labels after accounting for the possibility of pure chance (See Eq. 13). The range of values is -1 (complete disagreement) to + 1 (absolute agreement)^29^. The separability of the model’s predictions is measured by the Area Under the Receiver Operating Characteristic Curve (AUC), which shows the likelihood that the model will score a randomly selected positive instance higher than a randomly selected negative instance. It is a reliable statistic for evaluating the overall discriminative power and stability of the model’s confidence scores since it is threshold-invariant (See Eq. 16). These six quantitative performance measurements are computed using the following mathematical equations:

7 $$\:Overall\:Accuracy\:\left(OR\right)=\frac{\sum\:_{i=1}^{n}{TP}_{i}}{\sum\:_{i=1}^{n}{(TP}_{i}+{FP}_{i}+{TN}_{i}+{TN}_{i})}$$

8 $$\:Precision\:\left(Pr\right)=\:\sum\:_{i=1}^{n}\frac{{TP}_{i}+{FN}_{i}}{\sum\:_{j=1}^{n}{(TP}_{j}+{FN}_{j})}\times\:\frac{{TP}_{i}}{{TP}_{i}+{FP}_{i}}$$

9 $$\:Recall\:\left(Re\right)=\:\sum\:_{i=1}^{n}\frac{{TP}_{i}+{FN}_{i}}{\sum\:_{j=1}^{n}{(TP}_{j}+{FN}_{j})}\times\:\frac{{TP}_{i}}{{TP}_{i}+{FN}_{i}}$$

10 $$\:F1-Score\:\left(F1\right)=\:\sum\:_{i=1}^{n}\frac{{TP}_{i}+{FN}_{i}}{\sum\:_{j=1}^{n}{(TP}_{j}+{FN}_{j})}\times\:\frac{2\times\:{P}_{i}\times\:{R}_{i}}{{P}_{i}+{R}_{i}}$$

11 $$\:{P}_{i}=\frac{{TP}_{i}}{{TP}_{i}+{FP}_{i}}$$

12 $$\:{R}_{i}=\frac{{TP}_{i}}{{TP}_{i}+{FN}_{i}}$$

13 $$\:Kappa\:Score\:\left(KS\right)=\frac{{P}_{o}-{P}_{e}}{1-{P}_{e}}$$

14 $$\:{P}_{o}=\frac{\sum\:_{i=1}^{n}{TP}_{i}}{n}$$

15 $$\:{P}_{e}=\frac{({TP}_{i}+{FN}_{i})({TP}_{i}+{FP}_{i})}{{n}^{2}}$$

16 $$\:Area\:Under\:Curve\:\left(AUC\right)={\int\:}_{x=0}^{1}TPR\:\left({FPR}^{-1}\left(x\right)\right)\:dx$$

17 $$\:TPR=\frac{TP}{TP+FN}$$

18 $$\:FPR=\frac{FP}{FP+TN}$$

19 $$\:Final=\frac{F1+KS+AUC}{3}$$

The Final Score metric is the official evaluation criterion adopted by the OIA-ODIR benchmark^5^, computed as the arithmetic mean of F1-Score, Cohen’s Kappa Score, and AUC. This composite metric ensures a comprehensive and standardized assessment of model performance, accounting for class imbalance, classification reliability, and discriminative power simultaneously, and enables direct comparison with all prior work evaluated on this benchmark. The previous formulas are mostly compatible for the multiclassification task^30^. Where $\:n$ is the total number of classes, $\:i$ is the previously evaluated class, $\:j$ is all the classes when evaluating the total size of the dataset, $\:{TP}_{i}$ is the true positives for class $\:i$ (number of samples belonging to class $\:i$ that were correctly identified as class $\:i$). $\:{FP}_{i}$ is the false positives for class $\:i$ (number of samples from any class other than $\:i$ that were incorrectly labeled as class $\:i$). $\:{TN}_{i}$ is the true negatives for class $\:i$ (number of samples not belonging to class $\:i$ (i.e., from any of the other $\:n-1$ classes) that were correctly excluded from class $\:i$). $\:{FN}_{i}$ is the false negatives for class $\:i$ (number of samples actually belonging to class $\:i$ that were missed and incorrectly assigned to any class other than $\:i$). $\:{P}_{i}$ is the precision assigned to class $\:i$, and $\:Ri$ is the recall assigned to class $\:i$. $\:{P}_{o}$ is the proportion of observed agreement. It represents the proportion of samples for which the classifier’s prediction exactly matches the true ground truth label. $\:{P}_{e}$ or the Proportion of Expected Agreement, is the hypothetical agreement between the classifier and the ground truth that would be expected to happen at random, based only on the distribution (marginal probabilities) of the true labels and the predicted labels. $\:TPR$ is the true positive rate, also known as recall or sensitivity, calculates the percentage of real positive cases that the model accurately detected. $\:FPR$ is the false positive rate, it calculates the percentage of real negative cases that the model mistakenly classified as positive.

### Assessing the performance of various CNN architectures

A transfer learning technique is applied to resolve the addressed task of classifying various eye conditions. This required starting with a pre-trained CNN model, which turned out to be simpler and more efficient than initiating CNN training without prior weights. To enhance the pre-trained model’s performance, hyperparameters were adjusted. The ability of different pre-trained CNN models to function as a CNN base network for categorizing fundus photographs into eight different categories was assessed using the OIA-ODIR dataset. We combined the results from the left and right fundus images using the concatenation approach, which uses element-wise multiplication to reach the ultimate selection. Furthermore, we assessed how well the recommended image enhancement process worked to increase the CNN model’s ability to obtain more unique feature representations. Tables 2 and 3 show the categorization outcomes of several CNN base network on the on-site and off-site testing groups without the suggested pre-processing techniques and with them, respectively. These tables showed that all of the used CNN models performed substantially more effectively, yielding results that were between 8% and 19% better than those resulted without using the suggested preprocessing approach based on all performance metrics. The observed improvements stems from using enhanced photographs during DNN training, which facilitates the network’s ability to identify more distinguishing features of the lesions. Furthermore, this technique avoids skewing the training in favor of the majority classes.

### Ablation study

The following ablation experiments are designed to quantitatively justify the architectural complexity of the proposed DFD-Net system by isolating the individual contribution of each component, with particular focus on demonstrating the necessity of the dual-branch design over any single-branch alternative. To give a more thorough picture of the impact of each block in the suggested DFD-Net system, we perform ablation tests in this section. Table 4 represents the results from these tests. Test.1 relates to the ConvNeXt backbone, which serves as the CNN core of the suggested DFD-Net system and is trained using fundus images that have undergone necessary pre-processing steps. Using the on-site test set, this model scored an overall accuracy of 81.41%. In Test.2, the ConvNeXt model is replaced by the U-Net as the backbone model in the suggested DFD-Net system. The overall accuracy of Test.2 is improved by about 4%. Test.3 represents the fusion between the ConvNeXt model and the U-Net model resulting in another 4% increase in the overall accuracy due to the improved ability of the fusion between the two models to recognize fundus image lesions globally and locally. Finally, Test.4 referes to the proposed DFD-Net system, which encompasses a SENet block added to Test.3. A dense layer and a softmax activation layer use the collected feature represenations to create the likelihood distribution across eight different eye conditions. The proposed system represented in Test.4 has achieved OR, Pr, Re, F1, KS, and AUC values of 93.77%, 92.75%, 93.17%, 94.45%, 96.33%, and 99.87% using the on-site testing group, respectively. The highest test results can be attributed to learning the dependency between global and local attribute maps in the dual-branch system, which is part of the fusion mechanism used in the suggested system. As a result, these traits are reweighted, and more valuable information is retrieved.

**Table 2:** Classification scores of distinct CNN architectures without the suggested preprocessing technique using the on-site and off-site testing groups.

Backbones	On-site testing set						Off-site testing set					
	OR	Pr	Re	F1	KS	AUC	OR	Pr	Re	F1	KS	AUC
VGG16	45.69	45.88	44.40	45.66	50.42	73.55	42.93	43.24	44.35	43.78	51.16	70.08
VGG19	47.38	47.57	46.35	47.23	53.21	77.03	45.46	44.20	44.64	46.21	52.15	75.30
ResNet50	54.95	55.20	55.67	56.41	58.97	91.67	60.36	61.42	60.84	62.56	65.47	85.82
ResNet101	62.05	62.83	63.98	63.24	67.61	97.30	65.76	64.98	66.12	65.36	69.11	90.44
MobileNet	63.35	64.56	63.87	65.02	70.31	96.51	60.47	59.33	61.72	62.13	70.28	83.91
SE-Xception	63.44	64.21	64.79	65.18	71.70	96.28	64.58	64.60	65.88	63.25	71.49	91.42
HRNet	62.84	63.06	65.04	64.68	70.36	97.34	67.66	68.51	66.57	68.33	75.84	95.11
ConvNeXt	64.45	65.36	66.54	66.89	72.64	98.23	68.89	67.40	67.56	69.01	75.19	98.60
U-Net	64.13	65.41	66.98	67.14	72.51	98.10	70.28	71.13	69.39	71.83	76.58	98.92

Significant values are in bold.

**Table 3:** Classification scores of distinct CNN architectures with the suggested preprocessing technique using the on-site and the off-site testing groups.

Backbones	On-Site Testing Set						Off-Site Testing Set					
	OR	Pr	Re	F1	KS	AUC	OR	Pr	Re	F1	KS	AUC
VGG16	63.24	63.98	64.68	64.22	67.31	91.30	46.19	47.76	47.27	48.90	51.01	76.83
VGG19	65.56	66.08	65.91	67.30	68.56	93.04	50.27	49.17	48.80	49.18	53.59	80.12
ResNet50	67.23	66.35	67.78	67.12	68.55	94.21	67.58	68.60	66.39	68.71	75.34	95.12
ResNet101	75.56	76.34	76.31	75.18	78.54	96.41	70.79	71.27	72.39	71.40	75.18	96.19
MobileNet	71.36	70.40	70.09	71.28	73.66	95.93	68.40	67.83	69.70	69.25	70.59	95.48
SE-Xception	73.64	73.15	72.70	72.17	75.82	96.64	71.89	70.62	72.34	71.81	73.17	96.28
HRNet	79.33	80.41	79.50	80.13	83.22	98.11	72.15	73.80	72.37	74.72	76.55	97.27
ConvNeXt	81.41	80.30	80.13	81.87	83.78	98.26	75.47	74.18	76.29	77.50	79.59	97.34
U-Net	85.52	85.72	84.66	86.57	88.59	98.88	77.80	77.41	76.25	78.56	80.91	98.31

Significant values are in bold.

**Table 4:** The results of the ablation experiments of the suggested DFD-Net system using the on-site and off-site test groups.

Tests	On-Site Testing Set						Off-Site Testing Set					
	OR	Pr	Re	F1	KS	AUC	OR	Pr	Re	F1	KS	AUC
Test.1	81.41	80.30	80.13	81.87	83.78	98.26	80.90	79.91	81.68	81.59	86.63	98.02
Test.2	85.52	85.72	84.66	86.57	88.59	98.88	86.49	85.61	86.21	87.44	90.72	97.55
Test.3	89.82	90.13	90.59	91.46	94.12	99.20	90.12	91.30	91.49	89.91	94.67	99.23
Test.4	93.77	92.75	93.17	94.45	96.33	99.87	92.97	91.50	93.19	92.11	96.74	99.60

Significant values are in bold.

### Statistical analysis

A thorough statistical significance analysis was carried out on both the on-site and off-site test sets (each with $\:n=\mathrm{10,000}$) in order to thoroughly validate the improved performance of the suggested DFD-Net system and guarantee its superiority is statistically robust across various imaging environments. For each experimental framework, DFD-Net is evaluated independently against the nine baseline designs stated in Table 3. First, stratified performance measures were analyzed using bootstrapping to apply a parametric verification in order to evaluate global differences among classification correctness distributions. For both the on-site test set $\:\left(F\right(9.99990)=4854.2,\:P<0.001)$ and the off-site test set $\:\left(F\right(9.99990)=5124.7,\:P<0.001)$, the one-way Analysis of Variance (ANOVA)^31^ revealed extraordinarily large variance between the model groups, clearly rejecting the null hypothesis $\:\left({H}_{^\circ\:}\right)$. Tukey’s Honestly Significant Difference (HSD) post-hoc test^31^ was used to isolate certain pairwise differences. With a $\:P-value<0.001$ in each pairwise comparison for both datasets, the suggested DFD-Net demonstrated statistically significant superiority over all nine baselines. Even when compared to the best-performing baseline, U-Net, the performance improvement was still highly significant in both testing scenarios $\:P-value<0.001$. Second, DFD-Net and the baseline models were compared using McNemar’s test^32^ to examine paired categorical nominal decisions on identical picture vectors. In all paired tests, the resulting Chi-squared statistics rejected the marginal homogeneity of predictions with a $\:P-value<0.001$. The Statsmodels and SciPy Python libraries were used to create all computations and visualization profiles^33^. Lastly, bootstrap resampling was used to compute non-parametric 95% Confidence Intervals (CI). Table 5 shows the on-site and off-site test partitions, the 95% CI bounds for DFD-Net accuracy demonstrated zero overlap with any baseline intervals, offering conclusive empirical and mathematical evidence of the diagnostic improvement provided by the suggested framework.

**Table 5:** Comparative performance and statistical significance profile of the baseline architectures and the proposed DFD-Net on the on-site and off-site datasets.

Backbones	On-Site Test Set			Off-Site Test Set		
	OR	95% CI	*P*-value	OR	95% CI	*P*-value
VGG16	63.24	[62.29, 64.15]	< 0.001	46.19	[63.25, 65.13]	< 0.001
VGG19	65.56	[64.62, 66.46]	< 0.001	50.27	[49.29, 51.25]	< 0.001
ResNet50	67.23	[66.30, 68.12]	< 0.001	67.58	[66.66, 68.50]	< 0.001
ResNet101	75.56	[74.70, 76.38]	< 0.001	70.79	[69.90, 71.68]	< 0.001
MobileNet	71.36	[70.45, 72.23]	< 0.001	68.40	[67.49, 69.31]	< 0.001
SE-Xception	73.64	[72.76, 74.48]	< 0.001	71.89	[71.01, 72.77]	< 0.001
HRNet	79.33	[78.52, 80.11]	< 0.001	72.15	[71.27, 73.03]	< 0.001
ConvNeXt	81.41	[80.62, 82.16]	< 0.001	75.47	[74.62, 76.32]	< 0.001
U-Net	85.52	[84.81, 86.19]	< 0.001	77.80	[76.98, 78.62]	< 0.001
DFD-Net	93.77	[93.26, 94.24]	Reference	92.97	[92.41, 93.53]	Reference

Significant values are in bold.

## Comparison study

The suggested DFD-Net system’s efficiency was evaluated by contrasting its efficiency with the state-of-the-art techniques for categorizing various eye conditions. Table 6 displays the outcomes of the comparison. It is important to note that the state-of-the-art methodologies’ results in Table 5 are directly referenced from their original publications. This comparison is legitimate and in line with industry standards for evaluation because the OIA-ODIR dataset offers official, defined benchmark test sets that are the same for every study. To guarantee an even comparison, the developed DFD-Net system’s functionality was assessed, and When compared to other systems using criteria like the final score, which indicates their mean ideals (See Eq. 19), AUC, Cohen’s Kappa score, and F1-score. Feature maps is improved by integrating both global and local representations within a two-branch interactive design, facilitated by the use of a weighting technique, it is evident that the suggested DFD-Net system performs better than other current approaches in identifying various ocular diseases. Furthermore, we acquire multi-scale features to enhance the derived feature representations. As a result, the suggested DFD-Net system outperforms current techniques in identifying a variety of ocular disorders.

**Table 6:** An assessment between the suggested DFD-Net system and other state-of-the-art systems on the OIA-ODIR dataset on the on-site and off-site testing sets.

Methods	On-Site Testing Set				Off-Site Testing Set			
	Final score	F1	KS	AUC	Final score	F1	KS	AUC
VGG16 + SGD^7^	70.10	84.90	42.00	83.40	71.28	85.57	43.35	84.93
Inception-v4^5^	71.78	86.68	45.05	83.63	75.16	87.93	50.63	86.91
Vgg-16^5^	72.73	87.18	43.97	87.05	73.02	87.30	44.94	86.81
ResNet-101^34^	75.80	87.70	50.00	89.70	76.97	88.60	52.00	90.30
BFENet^35^	76.73	88.60	51.30	90.3	77.97	89.20	53.50	91.20
Fundus-DeepNet^2^	92.66	89.13	88.98	99.86	92.41	88.56	88.92	99.76
DSRA-CNN^19^	89.11	88.16	86.17	93.00	89.11	88.16	86.17	93.00
DFD-Net (our work)	96.88	94.45	96.33	99.87	96.15	92.11	96.74	99.60

Significant values are in bold.

**Fig. 8:**
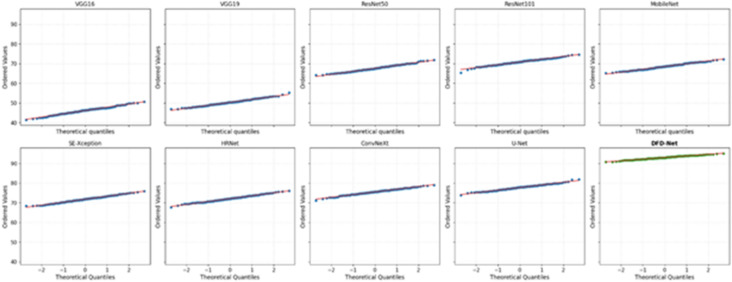
Q-Q plots of Bootstrap Overall accuracy on the On-site test set.

**Fig. 9:**
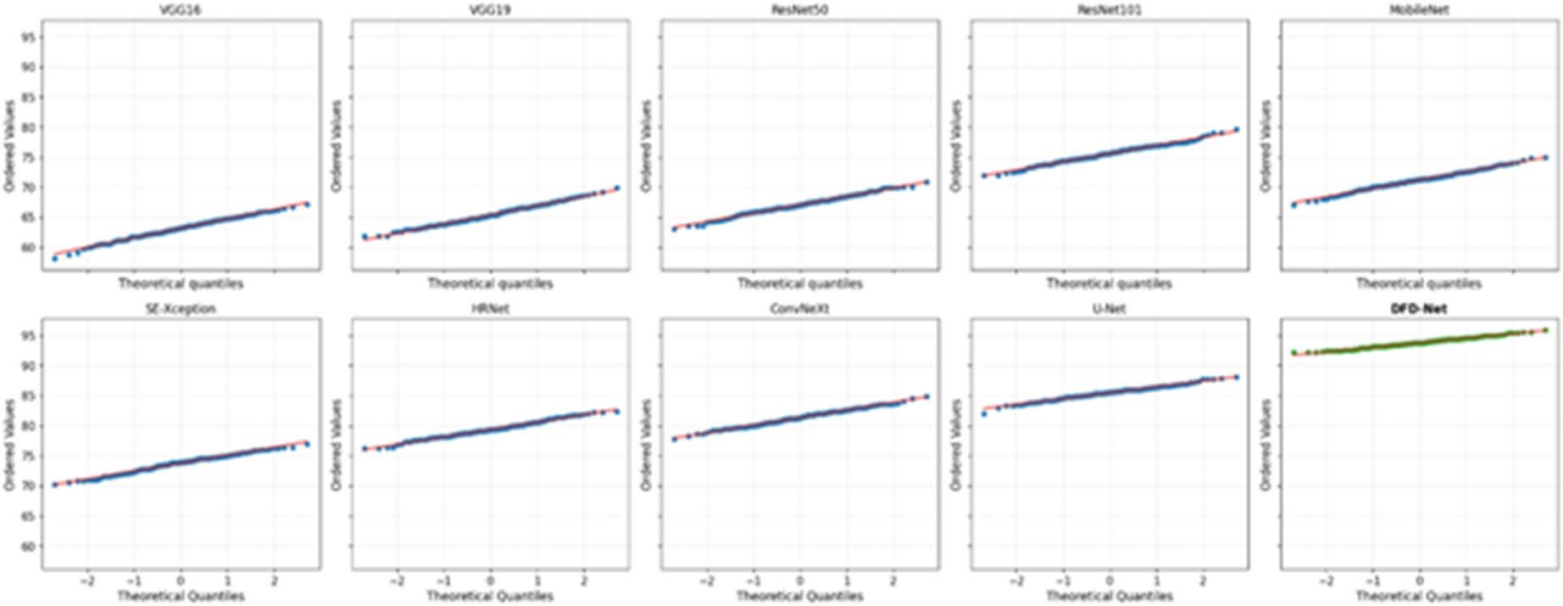
Q-Q plots of Bootstrap Overall accuracy on the Off-site test set.

The Quantile-Quantile (Q-Q) plots of the On-site and Off-site test sets, represented in Figs. 8 and 9, respectively, show the strict linear alignment of the sample quantiles against the red reference line across all ten models, visually verifying that the bootstrap accuracy scores follow a normal distribution, proving the graphical validity of our parametric assumptions.

**Fig. 10:**
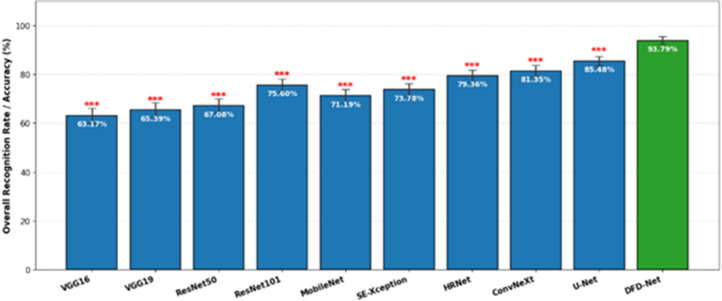
Overall comparison with 95% Bootstrap Confidence Intervals of the On-site test set.

**Fig. 11:**
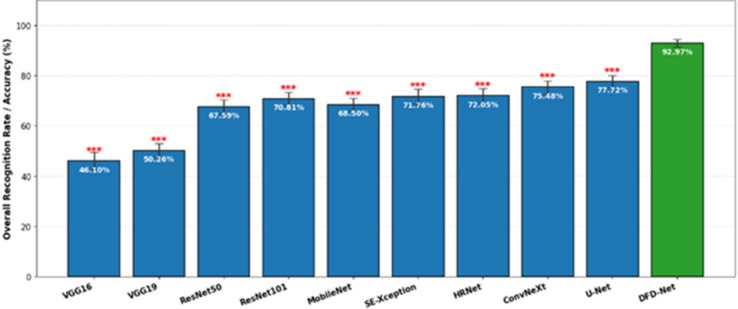
Overall comparison with 95% Bootstrap Confidence Intervals of the Off-site test set.

Second, the mean Overall Accuracy rate (OR%) and its exact 95% bootstrap confidence intervals (CI) are directly compared in Figs. 10 and 11 for the On-site and Off-site test sets, respectively. The consistent presence of significant markers (***) and the distinct vertical separation visually show that DFD-Net maintains a very different performance threshold over the baseline models in both imaging contexts.

**Fig. 12:**
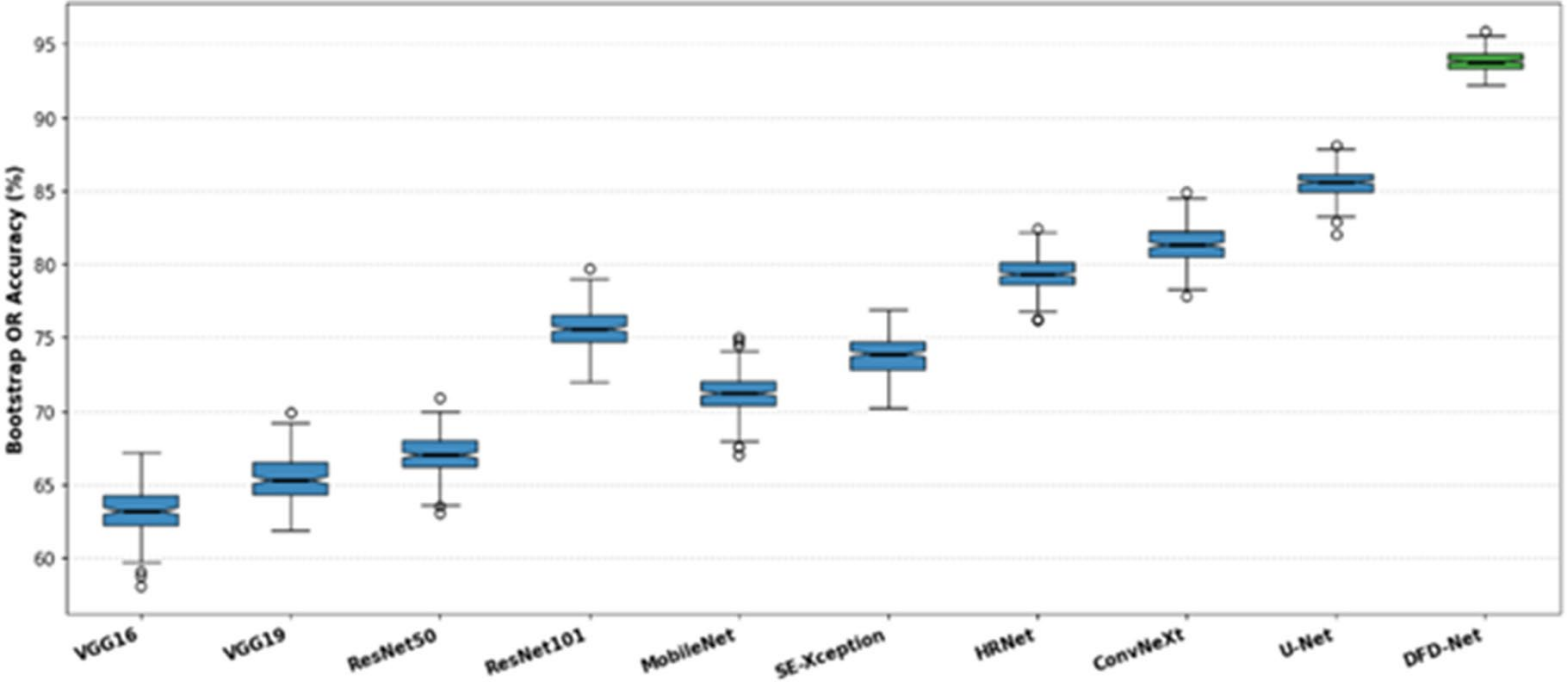
Box plots of Bootstrap overall accuracy distribution of the On-site test set.

**Fig. 13:**
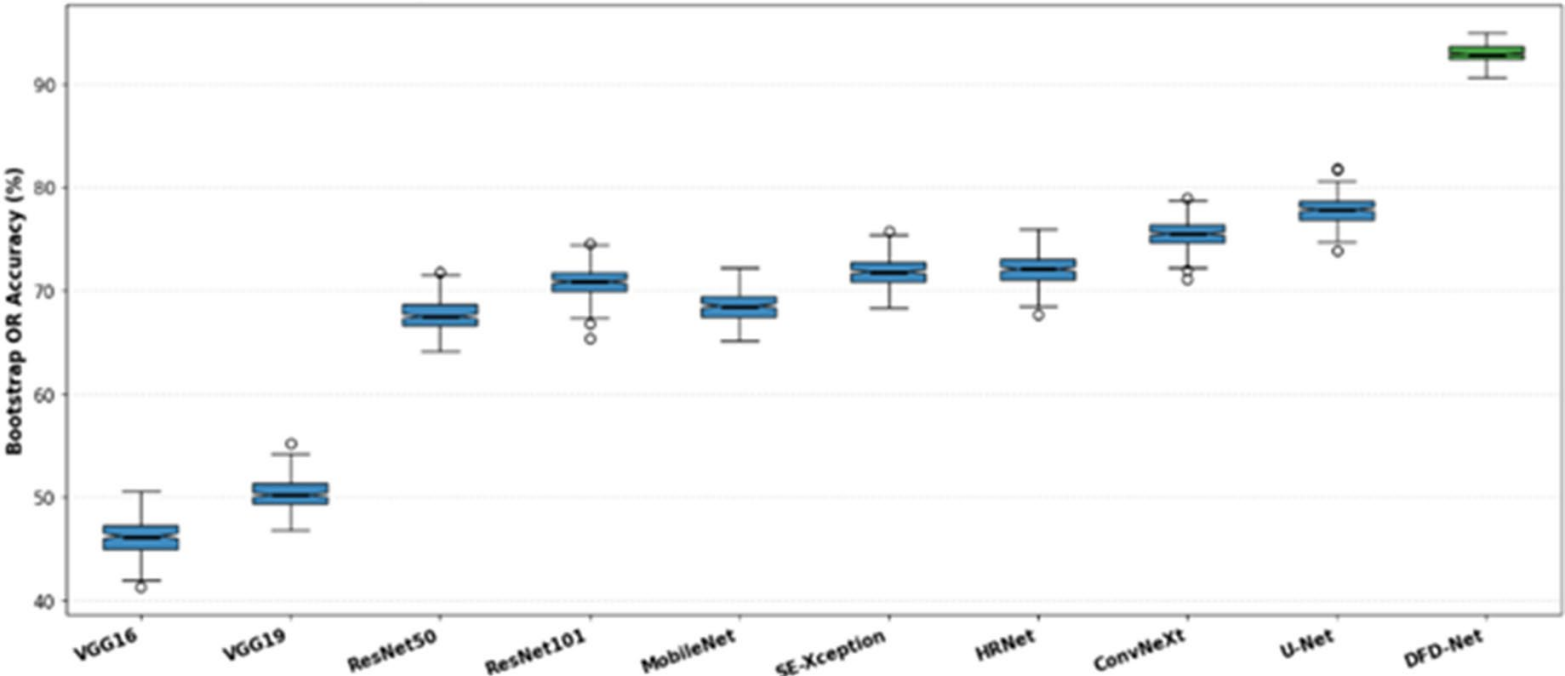
Box plots of Bootstrap overall accuracy distribution of the Off-site test set.

The notched box plots, which show the variance dispersion, interquartile ranges, and median shifts for each network, are shown in Figs. 12 and 13 for the On-site and Off-site test sets, respectively. Importantly, the notched boundary of the suggested DFD-Net stays totally isolated at the top of the spectrum even when the performance profiles of multiple baselines show different levels of graphical overlap. This complete absence of geometric overlap provides instantaneous visual confirmation of the remarkable generalization stability and strong diagnostic superiority provided by the suggested framework, particularly on the external Off-Site dataset.

**Fig. 14:**
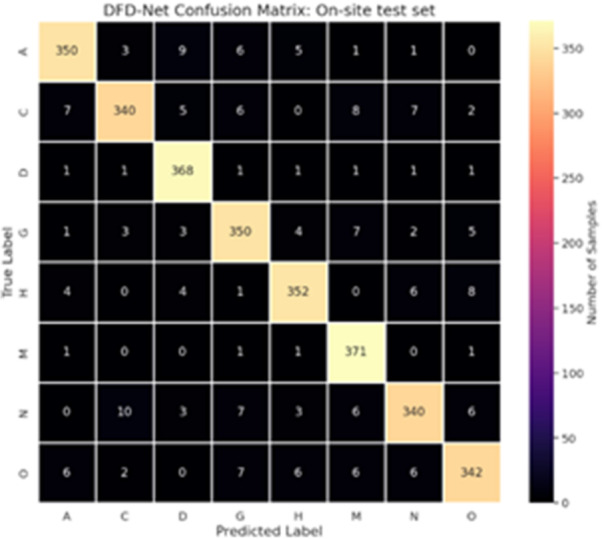
Confusion matrix showing the detection performance of the proposed DFF-Net system on the on-site test set.

**Fig. 15:**
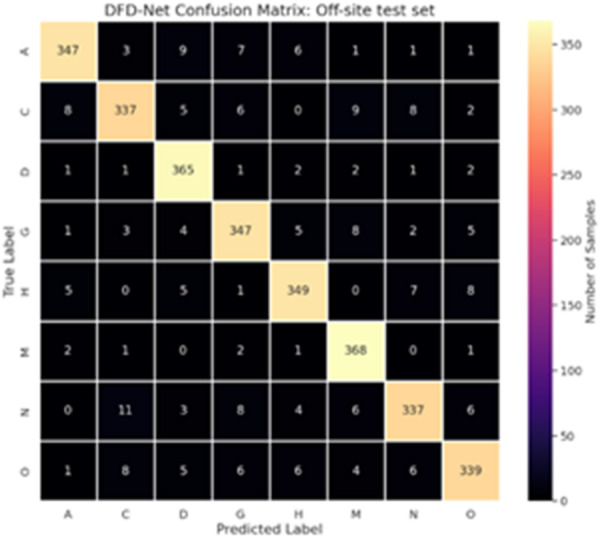
Confusion matrix showing the detection performance of the proposed DFF-Net system on the off-site test set.

By observing Figs. 14 and 15, we can confidently say that classes C, N, and O, which represent the cataract, normal, and other disease cases, scored lower than the other classes in their true positive rates, and that could be due to the blurry nature of the ‘Cataract’ fundus images. But for the ‘Normal’ class, this variance is explained by the model’s propensity to categorize borderline cases into disease groups as well as the inherent difficulty in characterizing the nuanced features of healthy eyes with large variability. In the context of clinical diagnosis, this tendency is maintained to guarantee great sensitivity in identifying the smallest abnormal indications, thus increasing the system’s overall accuracy. Finally, for the ‘Other diseases’ class, the low true positive rate stems primarily from its heterogeneity and poor definition, which challenges the fundamental assumptions of a classification model. It is remarkable that the RecallCE loss function, which dynamically amplifies minority class gradients to ensure high sensitivity in pathology detection, purposely results in the DFD-Net system’s predisposition to categorize borderline situations into illness categories rather than the Normal class. Because erroneous negatives in ocular screening are significantly more dangerous than false positives, this behavior is preferable from a therapeutic standpoint.

## Limitations

**Fig. 16:**
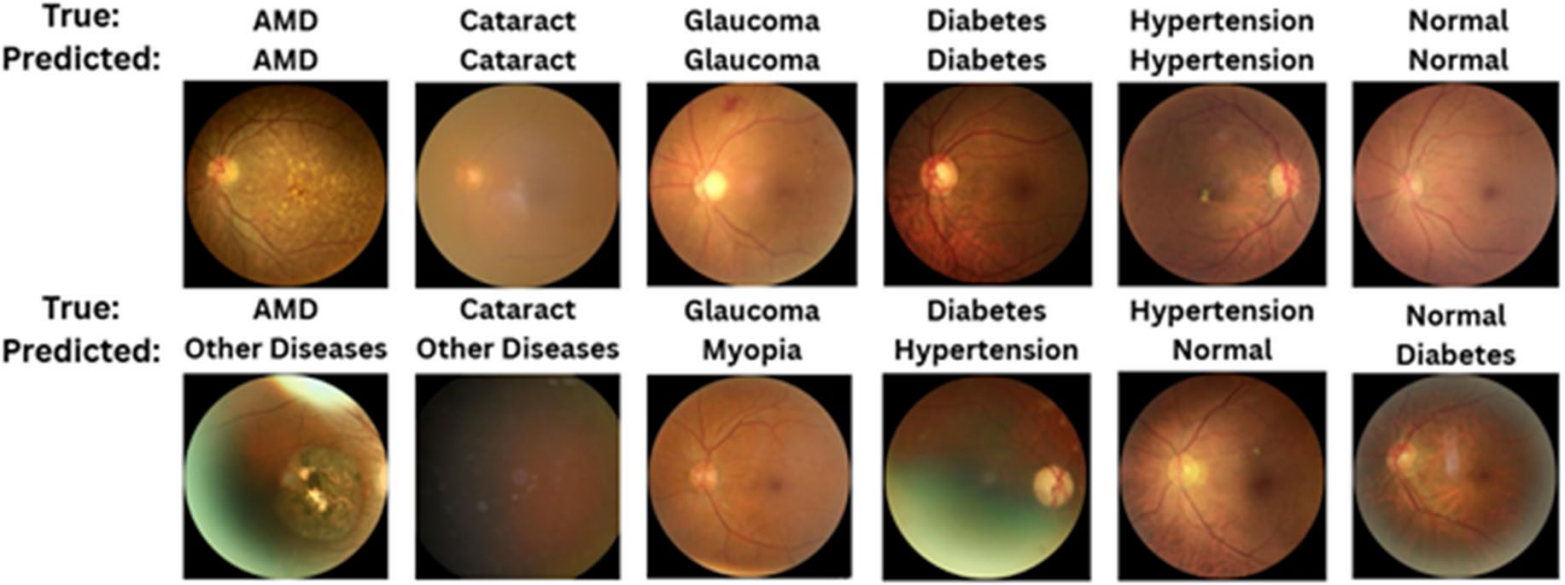
Samples accurately and inaccurately diagnosed across specific categories of eye conditions utilizing the suggested DFD-Net system.

Although the suggested DFD-Net system’s performance in correctly identifying several eye conditions from a pair of ocular photographs is encouraging. Even yet, to increase the accuracy of the suggested DFD-Net system, a few restrictions and difficulties must be resolved. The lack of available data is one of the primary challenges that could restrict the efficacy of the suggested method. It is well known that sufficient data is necessary for effective DNN training. In the scope of ocular disease classification, it may be challenging to locate labeled ocular photograph datasets that are adequately large and unique. Models may perform inadequately and overfit as a result of the lack of data. Additionally, it’s crucial to remember that the overall efficacy of the suggested approach may be significantly impacted by an uneven distribution of data among various categories of ocular diseases. Because the suggested approach may have trouble comprehending the characteristics of less prevalent diseases, this disparity may result in biased model predictions. However, by implementing a thorough data augmentation procedure on training in conditions linked to less prevalent diseases, this issue was rectified. Furthermore, rather than using the source images directly, the proposed DFD-Net architecture was trained utilizing the pre-processed ocular photographs. This strategy sought to reduce overfitting problems and generalization errors. Figure 16 displays a few examples of photographs that were successfully and mistakenly categorized in several categories of eye conditions.

## Conclusions and future work

By developing an effective, automated deep learning classification pipeline, the DFD-Net system effectively tackles the problem of multi-label ocular disease identification. The system’s three main parts are rigorous image preprocessing, a unique deep feature extraction module, and a multi-label classification head. It is intended to process bilateral pairs of fundus images. The system’s outstanding performance versus sophisticated current models was validated against the intricate OIA-ODIR dataset, which covers eight distinct ocular categories. The substantial effect of the suggested image enhancing process, which led to an average performance boost of between 8% and 19% across all assessment criteria used by the underlying CNN models, was a crucial discovery. Quantitatively, the system achieved consistently high scores regarding all performance metrics. For instance, in the on-site testing group, the proposed DFD-Net system scored final scores, F1 score, Kappa score, and AUC of 96,88%, 94.45%, 96.33%, and 99.87%, respectively. And on the off-site testing group, the system achieved slightly lower than the on-site testing group, scoring final scores, F1 score, Kappa score, and AUC of 96.15%, 92.11%, 96.74%, and 99.60%, respectively. These scores highlight the DFD-Net system’s efficacy in precisely identifying a variety of ocular conditions, providing substantial potential for promoting early ophthalmology diagnosis and treatment. The system’s ability to examine fundus image pairs from both eyes enhances the accuracy of disease detection by contributing to a thorough evaluation. Future research should focus on improving the system’s robustness and generalization by expanding the dataset to include a wider variety of representative ocular photographs, as well as evaluating the model across additional multi-class ocular disease datasets as they become publicly available, to further validate its robustness across different imaging devices, population demographics, and clinical settings. Additionally, by testing different deep learning models, finding novel weighting techniques, and refining hyper-parameters, the DFD-Net system’s overall accuracy can be further raised. Lastly, prospective studies can be conducted to verify the DFD-Net system’s effectiveness and practicality in practical situations, facilitating its future use in real-world clinical settings.

## Data Availability

The dataset is available at: https://github.com/nkicsl/OIA-ODIR.
